# Polypharmacy and Dynapenia in Older Adults Undergoing Rehabilitation After Fracture or Elective Orthopedic Surgery

**DOI:** 10.3390/medicina62010006

**Published:** 2025-12-19

**Authors:** Francesco Saverio Ragusa, Ligia J. Dominguez, Alessandro D’Aleo, Carlo Saccaro, Pasquale Mansueto, Nicola Veronese, Pietro Cataldo, Lee Smith, Mario Barbagallo

**Affiliations:** 1 Geriatric Unit, Department of Internal Medicine and Geriatrics, University of Palermo, 90127 Palermo, Italy; alessandro.daleo95@gmail.com (A.D.); carlosaccaro@gmail.com (C.S.); pasquale.mansueto@unipa.it (P.M.); mario.barbagallo@unipa.it (M.B.); 2 Department of Medicine and Surgery, Kore University of Enna, 94100 Enna, Italy; ligia.dominguez@unikore.it; 3 Faculty of Medcine, Saint Camillus International University of Health Sciences, 00131 Rome, Italy; nicola.veronese@unicamillus.org; 4 Sport and Exercise Sciences Research Unit, Department of Psychological, Pedagogical and Educational Sciences, University of Palermo, 90144 Palermo, Italy; pietro.cataldo@unipa.it; 5 Centre for Health Performance and Wellbeing, Anglia Ruskin University, Cambridge CB1 2LZ, UK; lee.smith@aru.ac.uk; 6 Department of Public Health, Faculty of Medicine, Biruni University, 34295 Istanbul, Turkey

**Keywords:** polypharmacy, dynapenia, muscle strength, older adults, rehabilitation, post-surgical patients, mortality, falls, hospitalization, frailty

## Abstract

*Background and Objectives*: Polypharmacy is common among older adults and its impact on the onset of dynapenia, reduced muscle strength and function, is largely unknown. Older adults hospitalized for either post-fracture or elective orthopedic surgery (knee, femur, or hip) and undergoing rehabilitation were included to investigate the association between polypharmacy and dynapenia. A further aim is to investigate associations between polypharmacy and dynapenia with outcomes including mortality, falls, and hospitalizations. *Materials and Methods*: On the fifth day following surgery, medical doctors administered a structured questionnaire along with physical and instrumental assessments. Polypharmacy was defined as the concurrent and regular use of 5 or more medications, dynapenia was assessed by measuring handgrip strength. The association between dynapenia and polypharmacy was detected with logistic regression, and their impact on adverse outcomes was assessed using Cox models, Kaplan–Meier curves and log-rank tests. *Results*: A total of 205 older adults (mean age 77.5 years; 79.5% women) were enrolled. After adjusting for sex, age, and the presence of multidimensional frailty, dynapenia was significantly associated with increased adverse outcomes such as mortality, falls, and hospitalizations (HR 2.96, 95% CI 1.22–7.20, *p* = 0.016). Similarly, polypharmacy was independently linked to a higher risk of mortality, falls and hospitalizations (HR 2.23, 95% CI 1.24–4.10, *p* = 0.007). At 6 months follow-up, polypharmacy showed a strong and significant association with dynapenia (adjusted OR 2.63, 95% CI 1.21–4.63, *p* = 0.019). *Conclusions*: These findings suggest that polypharmacy is strongly associated with dynapenia, both conditions are associated with adverse clinical outcomes in older hospitalized patients. Close monitoring and tailored interventions are recommended to mitigate these risks and improve rehabilitation outcomes in this vulnerable population.

## 1. Introduction

Aging is commonly accompanied by the onset of multiple chronic health conditions that require long-term management. To address these complex health needs, the use of several medications concurrently, commonly termed polypharmacy, often becomes necessary. Polypharmacy is defined as the regular use of 5 or more medications concurrently [1]. A Korean umbrella review of 295 studies and 59,552,762 participants showed how, globally, polypharmacy affects approximately 37% of the general population, with higher proportions observed among older adults (45%) and hospitalized patients (52%). Among frail older adults, the prevalence rises to 59%, reaching its peak in Europe (68%) [2]. Polypharmacy is linked to various adverse consequences, such as poor treatment adherence, increased risk of side effects, drug–drug and drug–disease interactions, falls, fractures, physical and cognitive decline, as well as medical errors [3].

Dynapenia, characterized by reduced muscle strength and function in older adults, is defined by handgrip strength (HGS) values below 27 kg for men and 16 kg for women [4], criteria later adopted by the European Working Group on Sarcopenia in Older People [5]. It typically begins in the fourth decade of life and accelerates significantly after the age of 65 years. It is estimated that approximately 50% of persons over 65 are affected, with prevalence rising to as high as 80% among those over 80 years [6]. Dynapenia develops through multiple mechanisms, including neuromuscular degeneration, hormonal decline, chronic inflammation, oxidative stress, mitochondrial dysfunction, and reduced physical activity [7]. Medications commonly prescribed for chronic diseases may further exacerbate muscle loss and weakness in older adults [8].

Polypharmacy has been strongly associated with dynapenia, given its well-documented association with adverse health outcomes and drug-related complications in older adults. However, current supporting this association remains limited. To the best of the authors’ knowledge, the only study specifically addressing this association is a Japanese investigation involving 257 community-dwelling older adults, which reported a significant association between dynapenia and polypharmacy [9]. Nonetheless, the study’s reliance on self-reported medication use and recruitment from senior welfare centers may have introduced selection bias by excluding frailer individuals.

The primary aim of the present study is to examine the association between polypharmacy and dynapenia in older patients hospitalized for rehabilitation following a fracture or elective orthopedic surgery, a population particularly vulnerable to adverse events. Additionally, the study aims to explore the association of dynapenia and polypharmacy with adverse outcomes, including mortality, falls, and rehospitalizations.

## 2. Materials and Methods

### 2.1. Population

This study was conducted using data collected at “Casa di Cura Clinica Latteri Valsalva” a Hospital in Palermo, Italy, between 1 January 2024 and 1 August 2024. Data collection was conducted in collaboration with medical post-graduate students of the Geriatric School within the Department of Internal Medicine and Geriatrics at the University of Palermo, Italy. The study received approval from the Ethics Committee “Comitato Etico Locale Palermo 1” of the University Hospital “Azienda Ospedaliera Universitaria Policlinico” (approval number: 07/2023, 12/12/23, approval date: 12 December 2023). Signed informed consent was obtained from all participants. For the purposes of this research, we included men and women aged over 60 years, who had undergone surgery for a femur fracture or received a prosthetic implant in the knee, femur, or hip on the fifth day after surgery and were hospitalized in the rehabilitation medicine department after a femur fracture or elective orthopedic surgery involving a prosthetic implant in the knee, femur, or hip. These represent common orthopedic interventions in older adults that require structured rehabilitation at “Casa di Cura Clinica Latteri Valsalva”. Data were collected at baseline and subsequently at 6 months. Exclusion criteria included were: age below 60 years, inability to understand or provide informed consent, or having undergone surgery more than five days prior to recruitment. Patients included in the study were administered a structured questionnaire and physical/instrumental testing by medical doctors on the fifth day after surgery. Specifically, surgical procedures encompassed internal fixation, joint replacement (prosthesis implant), reduction, and prosthesis replacement.

Rehabilitation was conducted according to usual clinical practice and supervised by physiotherapists and physicians specialized in physical medicine and rehabilitation, rather than following a predefined standardized protocol. This approach reflects routine clinical care and allows therapy to be individualized based on each patient’s functional status, tolerance, and rate of progression. For patients with hip prostheses who were unable to bear weight, rehabilitation followed a gradual progression, beginning with bed-based exercises, then sitting activities, verticalization, and assisted ambulation. As autonomy improved, patients transitioned from walking aids (e.g., walker to crutches) and progressed to gym-based strengthening and functional training. In the case of knee prostheses, rehabilitation included the use of a continuous passive motion device (Kinetec^®^, Aldershot, UK), with gradual increases of approximately 10° per day according to pain tolerance, up to a target of 110° of knee flexion, followed by muscular strengthening, range-of-motion recovery, balance training (e.g., bipodal stance on a balance board), and functional activities such as stair climbing.

The average duration of hospital-based rehabilitation was approximately two weeks. After discharge, patients were routinely referred to home-based physiotherapy services to ensure continuity of care and maintain functional gains. Given these individualized and stepwise interventions, no single standardized rehabilitation pathway was applied.

### 2.2. Polypharmacy

Polypharmacy was defined as the concurrent and regular use of 5 or more medications [1]. Data regarding number of medications were collected from medical records at baseline, and 6 months thereafter. Patients were categorized based on the number of regular medications into two groups: those taking fewer than five drugs and those taking five or more drugs, in accordance with the commonly used definition of polypharmacy. This grouping was used for analysis and is presented in Table 1 to examine the association between polypharmacy and dynapenia.

### 2.3. Dynapenia

HGS was assessed using a digital dynamometer (Kern & Sohn, GmbH, Balingen-Frommern, Germany), with all measurements performed by the same examiner to ensure consistency. Prior to testing, participants observed a demonstration and completed two practice trials. Following a five-minute familiarization period, they stood in an upright position with their arm, forearm, and wrist held in a neutral alignment. Participants were then instructed to apply maximum grip force for five seconds [10].

The test was performed three times using the dominant hand, with one-minute rest intervals between attempts. All three HGS values were recorded for statistical analysis, with the highest value used for evaluation. In accordance with current guidelines, dynapenia was defined as low HGS -below 27 kg for men and below 16 kg for women [4]. HGS data were collected at baseline and 6 months thereafter.

### 2.4. Surgical Procedures

Patients underwent a variety of orthopedic surgical procedures, including internal fixation, joint replacement (prosthesis implant), reduction, and prosthesis replacement. Internal fixation involved stabilization of fractures using plates, screws, or rods. Joint replacement consisted of implantation of a prosthetic device in the knee, femur, or hip to restore joint function. Reduction procedures entailed the realignment of displaced bone fragments, performed either closed or open depending on the fracture type. Prosthesis replacement referred to revision surgeries in which previously implanted prosthetic joints were replaced.

### 2.5. Participant Characteristics

The following variables were considered as potentially relevant covariates: sex, fracture, type of orthopedic surgery, Multidimensional Prognostic Index (MPI) [11], and the Geriatric Depression Scale (GDS) [12], all of which were treated as categorical variables. The MPI, derived from a Comprehensive Geriatric Assessment, was administered to all older patients at hospital admission. It includes eight domains:

1. Activities of Daily Living (ADL) [13]—basic daily care activities.
2. Instrumental Activities of Daily Living (IADL) [14]—more complex daily tasks.
3. Short Portable Mental Status Questionnaire (SPMSQ) [15]—cognitive status.
4. Cumulative Illness Rating Scale (CIRS) [16] Comorbidity Index—severity of comorbidities across 13 systems.
5. Mini Nutritional Assessment (MNA) [17]—nutritional status.
6. Exton Smith Scale [18]—risk of pressure sores.
7. Number of medications at discharge.
8. Cohabitation status—living with family, alone, or in an institution.

Each domain is scored as 0 (no problems), 0.5 (minor problems), or 1 (major problems). The mean score across domains gives the final MPI (0 = lowest risk, 1 = highest risk). Patients are classified into three risk groups: robust (<0.33), pre-frail (0.33–0.66), frail (>0.66).

Other variables, such as age, length of hospital stay, ADL, IADL, SPMSQ, CIRS, MNA, Exton Smith Scale, number of medications at discharge, Short Physical Performance Battery (SPPB), and gait speed, were treated as continuous variables.

Finally, use of antipsychotics/mood stabilizers, benzodiazepines/Z-drugs and antidepressants was registered.

### 2.6. Follow-Up

The same data, collected during the initial assessment, along with information on any adverse events (including death, falls, or hospitalizations), were recorded again at 6-month follow-up visits. For this follow-up, patients were first contacted by telephone (no questionnaires or data collection were carried out during the telephone call) and subsequently invited to return to “Clinica Latteri Valsalva”, where repeat physical and instrumental assessments were conducted. For time-dependent outcomes, patients were asked to provide the exact date of the event. Falls were assessed through participant or caregiver self-report during scheduled follow-up visits. Of the 205 patients initially enrolled in the study, 15 patients died during the follow-up period. Therefore, at the 6-month follow-up, 190 patients remained for assessment.

### 2.7. Statistical Analyses

Quantitative variables were summarized using means and standard deviations (SD), while categorical variables were presented as frequencies and percentages. Baseline characteristics of participants were compared according to antipsychotic or antidepressant use, employing Chi-square tests for categorical variables or Fisher’s exact test when expected cell counts were fewer than five.

The association between dynapenia and polypharmacy, with adverse outcomes, including all-cause mortality, falls, and hospitalizations during follow-up, was assessed using Cox proportional hazards models, with hazard ratios (HR) and 95% confidence intervals (CI) reported.

First, we performed univariate analyses for each variable. Variables meeting a *p*-value threshold of 0.10 were then entered into a multivariable model, along with adjustment factors, such as age, sex, and MPI. To evaluate multicollinearity, we calculated the Variance Inflation Factor (VIF). Predictors with VIF values above 2 were excluded, except for adjustment variables, which were retained regardless of collinearity. Survival curves were estimated using the Kaplan–Meier method, and differences between groups were compared with the log-rank test. The potential effect of polypharmacy on increasing the risk of dynapenia was further examined through logistic regression analysis and reporting data as odds ratios (ORs) with their 95% CIs. Negative events, such as mortality, falls and hospitalizations, were considered as composite outcome in the statistical analysis.

All statistical tests were two-sided, and a *p*-value < 0.05 was considered statistically significant. Analyses were conducted using RStudio (Version 2024.12.1+563).

## 3. Results

Of the 255 patients initially screened, 205 older adults (average age 77.5 years; 79.5% women) were enrolled in the study. The remaining 50 individuals were excluded due to severe dementia or inability to provide informed consent, with no available caregiver to assist.

### 3.1. Main Results

#### 3.1.1. Descriptive Characteristics

Table 1 shows the baseline characteristics of patients included. Participants were divided into two groups: less than 5 medications (no polypharmacy) (*n* = 52) and 5 or more medications (polypharmacy) (*n* = 153). At baseline, participants with polypharmacy were older (77.6 vs. 77.3 years, *p* = 0.005), had greater prevalence of frailty (32.0% vs. 17.3%, *p* < 0.001), higher CIRS scores (4.99 vs. 3.17, *p* < 0.001), lower MNA scores (9.10 vs. 9.65, *p* = 0.008), higher use of benzodiazepines (14.4% vs. 1.9%, *p* = 0.038), lower average HGS (16.6 kg vs. 19.0 kg, *p* = 0.045), and increased prevalence of dynapenia (50.3% vs. 38.5%, *p* < 0.001), compared to those without polypharmacy.

#### 3.1.2. Clinical Differences in Patients with and Without Fractures

Table 2 showed characteristics of patients with (*n* = 77) and without fractures (*n* = 127). Patients with fractures were older (80.7 vs. 75.6 years, *p* < 0.001) and had significantly longer hospital stays (20.4 vs. 16.5 days, *p* < 0.001) compared with those without fractures. They also showed higher frailty (54.5% vs. 12.5%, *p* < 0.001), lower functional status (ADL 1.51 vs. 3.96, *p* < 0.001; IADL 0.87 vs. 0.98, *p* = 0.01), and worse cognitive performance (SPSMQ 2.27 vs. 1.69, *p* = 0.02) compared with non-fractured patients. Significant differences were also observed in nutritional status (MNA 7.78 vs. 10.1, *p* < 0.001), GDS (severe 45.4% vs. 19.5% *p* < 0.001), physical performance (SPPB 0.78 vs. 2.10 *p* < 0.001, gait speed 0.40 vs. 0.94 *p* < 0.001), handgrip strength (HGS 15.5 vs. 18.2 kg, *p* = 0.01).

#### 3.1.3. Dynapenia and Adverse Outcomes

During the 6 months follow-up, we documented 22 fall events, 15 deaths, and 12 hospitalizations. Table 3 shows that dynapenia significantly increased the likelihood of adverse outcomes, such as mortality, falls, or hospitalizations (HR 5.61, 95% CI 2.51–12.57, *p* < 0.001). This association remains significant after adjusting for potential confounders, including sex, age, and MPI (HR 2.96, 95% CI 1.22–7.20, *p* = 0.016). Similarly, Table 3 shows that polypharmacy is also associated with a higher risk of these outcomes (HR 2.34, 95% CI 1.20–4.30, *p* < 0.001). This association remained significant after adjustment for the same confounders (HR 2.23, 95% CI 1.24–4.10, *p* = 0.007).

**Figure 1:**
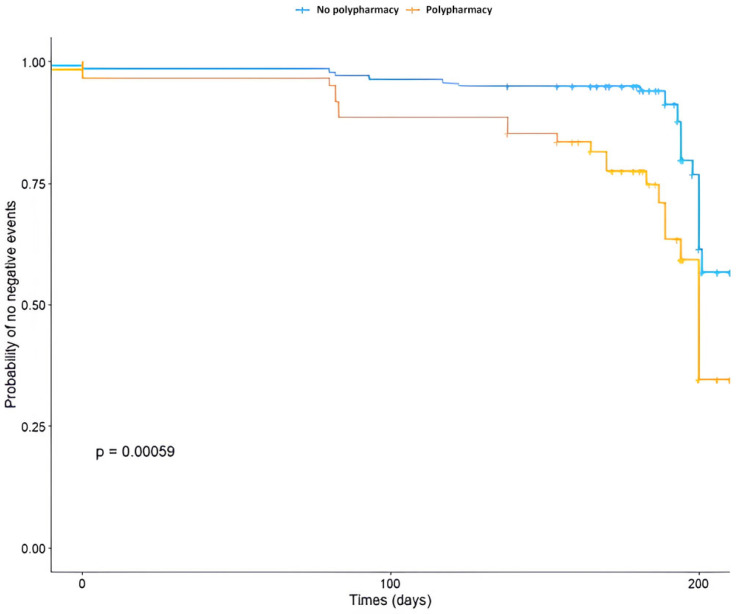
Probability of negative events (mortality, falls, hospitalizations) for patients with polypharmacy against patients without polypharmacy.

**Figure 2:**
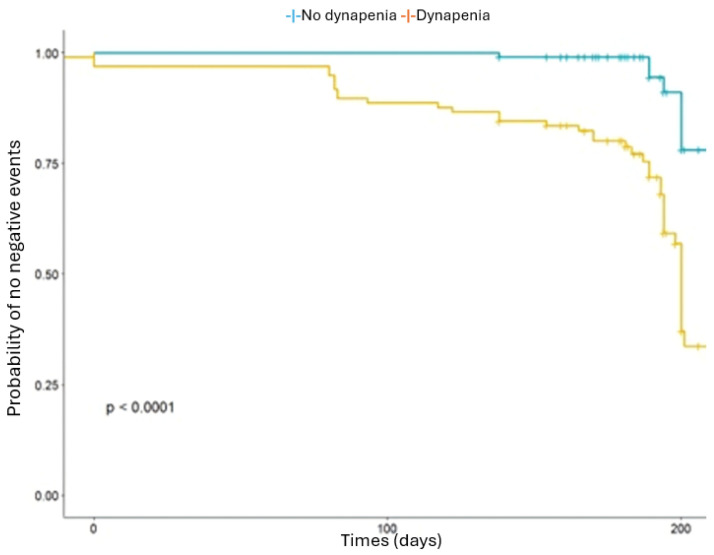
Probability of negative events (mortality, falls, hospitalizations) for patients with dynapenia against those without dynapenia.

Figure 1 and Figure 2 present survival curves. Figure 1 compares risk of adverse outcomes based on polypharmacy against non-polypharmacy (log-rank test *p* = 0.00059), while Figure 2 compares individuals with dynapenia to those without dynapenia (log-rank test *p* < 0.0001).

#### 3.1.4. Polypharmacy and Dynapenia

**Figure 3:**
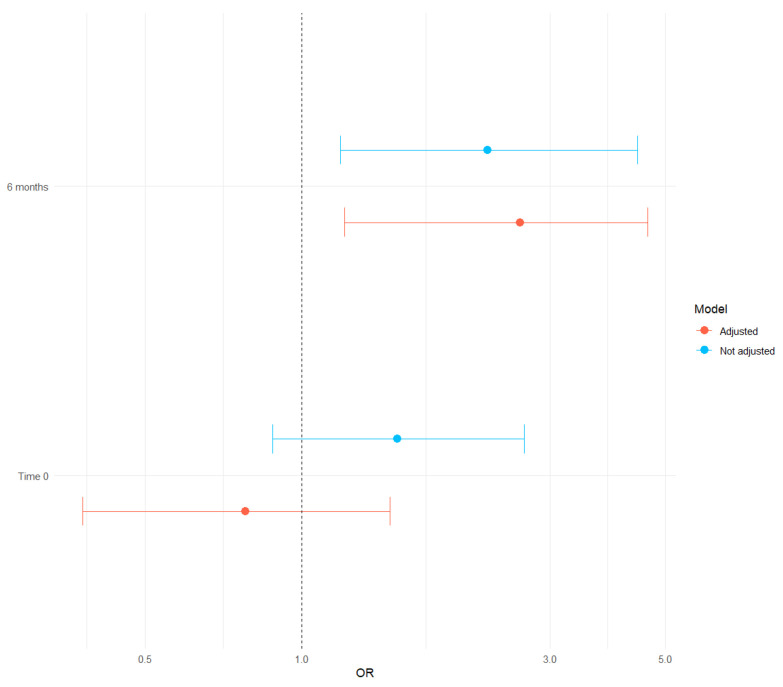
Forest plot for association between polypharmacy with the presence of dynapenia at times 0 and 6 months (adjusted and unadjusted models).

Figure 3 presents the association between polypharmacy with the presence of dynapenia at times 0 and 6 months. At baseline, the unadjusted OR is 1.53 (95% CI 0.88–2.68, *p* < 0.13) and the adjusted OR is 0.78 (95% CI 0.38–1.48, *p* = 0.43). At 6 months, there was a very strong and significant association, with an unadjusted OR of 2.28 (95% CI 1.19–4.42, *p* = 0.013) and an adjusted OR of 2.63 (95% CI 1.21–4.63, *p* = 0.019) (Table 4). At baseline, 74.63% of patients were classified as having polypharmacy (≥5 medications), which decreased to 60.21% at the 6-month follow-up.

Table 5 shows the HR of negative outcomes for the use of specific medications commonly prescribed in older adults. Compared with participants not using these medications, the use of antipsychotics/mood stabilizers was associated with a more than threefold increased risk of these negative events (HR = 3.27, 95% CI 1.43–7.50, *p* = 0.005), while benzodiazepines/Z-drugs were associated with nearly a threefold increased risk (HR = 2.93, 95% CI 1.27–6.74, *p* = 0.012). No significant association was observed for antidepressant use (HR = 1.87, 95% CI 0.44–7.91, *p* = 0.392).

#### 3.1.5. Specific Medications and Negative Outcomes

**Figure 4:**
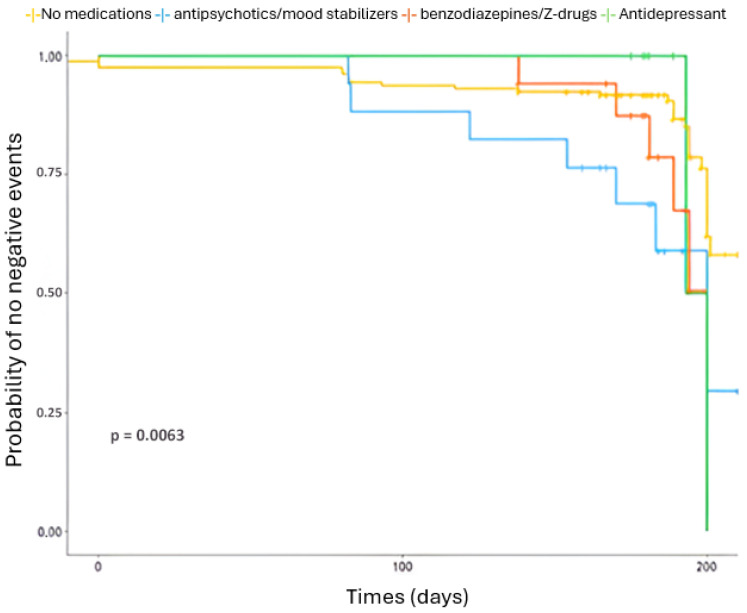
Probability of negative events (mortality, falls, hospitalizations) of patients taking specific medication in older people.

Figure 4 illustrate the association between the use of specific medications and negative events: Kaplan–Meier curves showed a significantly lower survival probability for participants using antipsychotics/mood stabilizers or benzodiazepines/Z-drugs compared with non-users (log-rank test *p* = 0.006).

## 4. Discussion

To the best of our knowledge, this is one of the first studies examining the association of dynapenia and polypharmacy in older adults hospitalized for either post-fracture or elective orthopedic surgery (knee, femur, or hip) and undergoing rehabilitation. The primary aim was to assess this association in patients undergoing internal fixation, joint replacement (prosthesis implant), reduction or prosthesis replacement. A secondary aim was to evaluate whether dynapenia and polypharmacy predict adverse outcomes, such as mortality, falls, and hospital readmissions. Given the observational design, the findings should be interpreted as associative rather than causal, and no causal inferences can be drawn from the observed association.

Compared with those without polypharmacy at baseline, participants with polypharmacy were older, showed greater prevalence of frailty, higher CIRS scores, lower MNA scores, significantly greater use of benzodiazepines/Z-drugs, lower average HGS, and higher frequency of dynapenia. The present findings support those of previous literature. For example, a study, carried out in Italy on 4402 participants, found that the incidence of frailty was approximately double in those taking 4–6 medications and 6 times higher in people taking ≥7 compared with those taking 0–3 medications [19]. Moreover, an American study of 4666 patients observed a negative association between HGS and polypharmacy in older people [20]. Finally, a study, carried out in Norway on 270 participants, revealed that polypharmacy was associated with higher odds of malnutrition risk, measured with MNA [21].

Dynapenia was significantly associated with increased likelihood of adverse outcomes, such as mortality, falls, and hospitalizations, after adjusting for potential confounders, including sex, age, and MPI. To the best of our knowledge, no previous research has specifically analyzed the effects of dynapenia on these negative outcomes. Former studies have examined dynapenia focused solely on a single adverse outcome or alongside other factors or focused solely on a single adverse outcome. For instance, a study, conducted in Iran involving 1354 participants, examined dynapenia in conjunction with abdominal obesity and found that dynapenia alone-without abdominal obesity-was linked to a higher risk of mortality [22]; a Japanese study on 661 participants undergoing hemodialysis found that dynapenia was associated with increased risks of all-cause mortality and cardiovascular hospitalizations [23]. Another study from Great Britain, involving 4239 participants, assessed the impact of dynapenia and abdominal obesity on fall risk. It found a synergistic effect of dynapenia and obesity on falls risk among men, but not in women [24].

In the present study, polypharmacy was associated with a higher risk of negative events, such as mortality, falls, and hospitalizations, and this association remained significant after adjustment for potentially relevant confounders. These findings also support previous literature. For example, a study from Korea involving 3,007,620 older adults found that polypharmacy was associated with a 25% higher risk of mortality and a 18% higher risk of hospitalizations [25]. Moreover, a study, conducted in England on 5213 older adults, showed that nearly one-third of participants were taking five or more medications, which was significantly associated with a 21% higher rate of falls over a two-year period [26].

In this study, we included older adults hospitalized for either post-fracture or elective orthopedic surgery (knee, femur, or hip) and undergoing rehabilitation These represent common orthopedic interventions in older adults [27] that require structured rehabilitation. The study was conducted in collaboration with a clinic that performs predominantly these types of procedures, under a formal agreement with our Department of Internal Medicine and Geriatrics at the University of Palermo.

Dynapenia is a multifactorial condition driven by both internal and external factors. Aging leads to neuromuscular changes, hormonal decline, and especially chronic low-grade inflammation, which promotes muscle catabolism and impairs repair mechanisms [7]. Oxidative stress, caused by an excess of reactive oxygen species, damages proteins, lipids, and DNA, further reducing muscle function, while mitochondrial dysfunction decreases energy production and worsens fatigue [28]. External contributors, such as physical inactivity, medication side effects, and poor nutrition, exacerbate these processes [29]. Altogether, these mechanisms drive impaired protein synthesis and muscle atrophy, and enhance loss of strength, which can explain why dynapenia is strongly linked to adverse outcomes, such as falls, hospitalizations, and even increased mortality [30].

After a 6 months follow-up, we found that polypharmacy was significantly and strongly associated with an increased presence of dynapenia. Polypharmacy is associated with dynapenia through multiple mechanisms. Medications, such as statins and glucocorticoids have direct catabolic or toxic effects on muscle tissue, impairing protein synthesis and accelerating atrophy [31,32]. Although glucocorticoids and statins were not included in the present analysis, these medications are also widely prescribed in older adults [33]. Given their high prevalence in polypharmacy regimens, many patients are likely to be taking at least one of these medications, which may contribute to muscle weakness and functional decline. This should be considered when interpreting the broader implications of medication burden in this population. It was also showed how psychotropic medications may reduce neuromuscular activation, leading to decreased muscle strength and slowed motor response [34]. In addition, some psychotropic agents are associated with sedation, reduced physical activity, and fatigue, which can further accelerate muscle deconditioning and functional decline over time [35]. Drug–drug interactions and side effects, such as fatigue, dizziness, or nutrient malabsorption, may decrease physical activity and worsen nutritional status, further weakening muscle strength [36]. Altogether, these factors highlight the association between polypharmacy and dynapenia in older adults.

These findings indicate that polypharmacy is highly prevalent among older adults undergoing orthopedic rehabilitation and is strongly associated with dynapenia and adverse clinical outcomes. The observed reduction in polypharmacy over 6 months suggests that medication management during and after hospitalization may influence these risks. Monitoring and optimizing medication regimens in this population could therefore be an important strategy to reduce frailty-related complications and improve rehabilitation outcomes.

It is important to note that the biological impact of polypharmacy is not solely determined by the number of medications prescribed, but also by the qualitative drug burden. It refers to the type and pharmacological characteristics of the medications a person takes, rather than the number of medications alone. It considers how specific drug classes, mechanisms of action, side-effect profiles, and potential drug–drug interactions contribute to the overall physiological and functional impact of treatment [37]. Qualitative drug burden influences physical, psychological, social, and financial wellbeing, and how patients continually attempt to adapt their routines to manage the demands of their treatment [38].

We also found that the use of antipsychotics, mood stabilizers, benzodiazepines, and Z-drugs was linked to a markedly higher likelihood of adverse events, such as mortality, falls, and hospitalizations. In contrast, antidepressant use did not show a significant association with such outcomes. A study conducted in Sweeden including 1,288,875 older people reported how antidepressants emerged as the psychotropic medications most strongly associated with an increased risk of fall-related injuries, while antipsychotics were more closely linked to higher rates of hospitalizations and mortality [39]. These findings suggest that different classes of psychotropic medications may contribute to adverse outcomes through distinct mechanisms. Antidepressants, particularly those with sedative or hypotensive effects, could increase vulnerability to falls and related injuries [40]. Conversely, antipsychotics may be linked to more severe outcomes, such as hospitalizations and mortality, possibly due to their metabolic, cardiovascular, or neurological side effects [41], as well as the fact that they are often prescribed to patients with more complex clinical profiles.

Given the observational design, causal relationships cannot be established. Reverse causation is plausible, as patients with lower muscle strength or greater comorbidity burden may be more likely to receive multiple medications. The absence of preoperative handgrip strength data further limits causal inference. Therefore, our findings should be interpreted as demonstrating associations rather than causality and are primarily hypothesis-generating. Future longitudinal studies with preoperative assessments and causal modeling are needed to clarify these relationships.

This study should be viewed considering both its strengths and its limitations. To our knowledge, it represents one of the first attempts to compare dynapenia and polypharmacy following surgical treatment, analyzing their possible negative consequences. In addition, the six-month observation period provided the opportunity to monitor changes over time and to capture both improvement and decline in patients’ health status.

Several limitations must be considered when interpreting these findings. First, the assessment was performed only after surgery, without preoperative data that would have provided a clearer baseline, raising the possibility of reverse causation. The inclusion of different fracture types and orthopedic procedures, combined with the absence of detailed perioperative variables (e.g., surgical severity, blood loss, anesthesia type, analgesia regimen), introduces heterogeneity that may confound postoperative recovery and muscle strength, so the results should be interpreted with caution. Handgrip strength was measured shortly after surgery and could have been influenced by pain or restricted mobility, although the procedures involved the lower limbs. Rehabilitation exposure was not quantified (e.g., therapy hours, ambulation milestones, weight-bearing status), leaving variability in treatment dose as an uncontrolled confounder. Conducting the study in a single institution with a relatively small sample limits generalizability and statistical power, and the exclusion of patients with severe dementia or without caregivers may have introduced selection bias. Hospitalizations and falls outside the institution were based on self-report, which may have led to under-ascertainment of minor events. Because mixed-effects or other longitudinal models were not used, within-subject correlation across repeated measurements may not be fully accounted for. Finally, we did not adjust our data for specific medication classes or doses, leaving the potential influence of high-impact drugs unmeasured.

Future multicenter studies with larger and more diverse populations are needed, incorporating standardized pre- and postoperative assessments and comprehensive evaluation of polypharmacy and overall drug burden. Such research would clarify the complex relationship between medication use and postoperative functional outcomes and provide robust evidence to guide clinical interventions and optimize rehabilitation strategies in older adults undergoing orthopedic surgery. At the same time future studies should incorporate standardized or measurable rehabilitation metrics to better account for this source of heterogeneity.

## 5. Conclusions

In conclusion, this study suggests that polypharmacy is strongly associated with dynapenia, and both conditions are linked to adverse outcomes, including mortality, falls, and hospitalizations in older adults after surgery. These findings highlight the importance of careful medication management and targeted interventions during rehabilitation to improve patient outcomes.

## Figures and Tables

**Table 1:** Characteristics of patients included at baseline.

	Medications < 5 (*n* = 52)	Medications ≥ 5 (*n* = 153)	Overall (*n* = 205)	*p*-Value
**Sex** (N, %)				
F	39 (75.0%)	124 (81.0%)	163 (79.5%)	0.85
**Age (years)**				
Mean (SD)	77.3 (7.48)	77.6 (6.79)	77.5 (6.96)	**0.005**
**Length of stay (days)**				
Mean (SD)	18.3 (4.37)	17.9 (4.80)	18.0 (4.69)	0.65
**Fractures**				
N (%)	22 (42.3%)	56 (36.6%)	78 (38%)	**0.003**
**Orthopedic surgery** (N, %)				
Internal fixation	13 (25.0%)	38 (24.8%)	51 (24.9%)	0.46
Joint replacement (prosthesis implant)	34 (65.4%)	107 (69.9%)	141 (68.8%)	
Reduction (Fracture realignment)	3 (5.8%)	1 (0.7%)	4 (1.9%)	
Prosthesis replacement	2 (3.8%)	7 (4.6%)	9 (4.4%)	
**MPI** (N, %)				
Robust	7 (13.5%)	6 (3.9%)	13 (6.3%)	**<0.001**
Pre-frail	36 (69.2%)	98 (64.1%)	134 (65.4%)	
Frail	9 (17.3%)	49 (32.0%)	58 (28.3%)	
**ADL**				
Mean (SD)	3.13 (1.84)	3.00 (1.86)	3.03 (1.85)	0.22
**IADL**				
Mean (SD)	0.923 (0.269)	0.941 (0.236)	0.937 (0.244)	0.18
**SPSMQ**				
Mean (SD)	1.96 (1.92)	1.90 (1.59)	1.91 (1.68)	0.37
**CIRS**				
Mean (SD)	3.17 (0.834)	4.99 (1.07)	4.53 (1.29)	**<0.001**
**MNA**				
Mean (SD)	9.65 (2.24)	9.10 (2.31)	9.24 (2.30)	**0.008**
**Exton Smith scale**				
Mean (SD)	13.4 (2.89)	12.6 (2.88)	13.1 (2.90)	0.08
**Medications**				
Mean (SD)	3.35 (0.764)	7.41 (2.16)	6.38 (2.60)	**<0.001**
**Living status** (N, %)				
Family	30 (57.7%)	87 (56.9%)	117 (57.1%)	**0.028**
Alone	22 (42.3%)	64 (41.8%)	86 (42.0%)	
Institute	0 (0%)	2 (1.3%)	2 (1.0%)	
**GDS** (N, %)				
0	9 (17.3%)	21 (13.7%)	30 (14.6%)	0.44
1–2	28 (53.8%)	88 (57.5%)	116 (56.6%)	
≥3	15 (28.8%)	44 (28.8%)	59 (28.8%)	
**SPPB**				
Mean (SD)	1.67 (1.40)	1.58 (1.86)	1.60 (1.75)	0.20
**Gait Speed (m/sec)**				
Mean (SD)	0.788 (0.750)	0.724 (0.929)	0.740 (0.886)	0.49
**Antipsychotics/mood stabilizers**				
N (%)	4 (7.7%)	13 (8.5%)	17 (8.3%)	0.25
**Benzodiazepines/Z-drugs**				
N (%)	1 (1.9%)	22 (14.4%)	23 (11.2%)	**0.038**
**Antidepressants**				
N (%)	1 (1.9%)	17 (11.1%)	18 (8.8%)	0.16
**Mortality**				
N (%)	4 (7.7%)	11 (7.2%)	15 (7.3%)	0.19
**Falls**				
N (%)	4 (7.7%)	18 (11.8%)	22 (10.7%)	0.30
**Hospitalizations**				
N (%)	1 (1.9%)	10 (6.5%)	11 (5.4%)	0.76
**HGS (kg)**				
Mean (SD)	19.0 (8.08)	16.6 (6.69)	17.2 (7.12)	**0.045**
**Dynapenia**				
Mean (SD)	20 (38.5%)	77 (50.3%)	97 (47.3%)	**<0.001**

Abbreviations (in alphabetic order): ADL = Activities of Daily Living, CIRS = Cumulative Illness Rating Scale, GDS = Geriatric Depression Scale, HGS = Handgrip Strength; IADL = Instrumental Activities of Daily Living, MNA = Mini Nutritional Assessment, MPI = Multidimensional Prognostic Index, SD: Standard Deviation, SPPB = Short Physical Performance Battery, SPSMQ = Short Portable Mental Status Questionnaire. Bold font is used to highlight statistically significant *p*-values (*p* < 0.05).

**Table 2:** Characteristics of patients included with and without fractures.

	No Fractures (*n* = 127)	Fractures (*n* = 77)	Overall (*n* = 205)	*p* Values
**Sex**				
F	107 (83.6%)	56 (72.7%)	163 (79.5%)	0.09
**Age (years)**				
Mean (SD)	75.6 (5.35)	80.7 (8.13)	77.5 (6.96)	**<0.001**
**Length of stay (days)**				
Mean (SD)	16.5 (3.33)	20.4 (5.59)	18.0 (4.69)	**<0.001**
**MPI** (N, %)				
Robust	11 (8.6%)	2 (2.6%)	13 (6.3%)	**<0.001**
Pre-frail	101 (78.9%)	33 (42.9%)	134 (65.4%)	
Frail	16 (12.5%)	42 (54.5%)	58 (28.3%)	
**ADL**				
Mean (SD)	3.96 (1.37)	1.51 (1.50)	3.03 (1.85)	**<0.001**
**IADL**				
Mean (SD)	0.98 (0.15)	0.87 (0.34)	0.937 (0.244)	**0.01**
**SPSMQ**				
Mean (SD)	1.69 (1.47)	2.27 (1.93)	1.91 (1.68)	**0.02**
**CIRS**				
Mean (SD)	4.62 (1.29)	4.39 (1.27)	4.53 (1.29)	0.24
**MNA**				
Mean (SD)	10.1 (1.89)	7.78 (2.19)	9.24 (2.30)	**<0.001**
**Medications**				
Mean (SD)	6.27 (2.36)	6.53 (2.98)	6.38 (2.60)	0.50
**Living status** (N, %)				
Family	74 (57.8%)	43 (55.8%)	117 (57.1%)	0.94
Alone	53 (41.4%)	33 (42.9%)	86 (42.0%)	
Institute	1 (0.8%)	1 (1.3%)	2 (1.0%)	
**GDS**				
mild-moderate	82 (64.1%)	33 (42.9%)	115 (56.1%)	**<0.001**
absent	21 (16.4%)	9 (11.7%)	30 (14.6%)	
severe	25 (19.5%)	35 (45.4%)	60 (29.3%)	
**SPPB**				
Mean (SD)	2.10 (1.78)	0.78 (1.35)	1.60 (1.75)	**<0.001**
**Gait Speed (m/sec)**				
Mean (SD)	0.94 (0.92)	0.40 (0.71)	0.740 (0.886)	**<0.001**
**Antipsychotics/mood stabilizers**				
N (%)	7 (5.5%)	10 (13.0%)	17 (8.3%)	0.10
**Benzodiazepines/Z-drugs**				
N (%)	9 (7.1%)	14 (18.2%)	23 (11.2%)	**0.02**
**Antidepressants**				
N (%)	7 (5.5%)	11 (14.3%)	18 (8.8%)	0.06
**Dynapenia**				
Mean (SD)	46 (36.2%)	51 (66.2%)	0.473 (0.501)	**<0.001**
**Polypharmacy**				
N (%)	98 (77.1%)	55 (71.4%)	153 (74.6%)	**0.003**
**HGS (kg)**				
Mean (SD)	18.2 (5.91)	15.5 (8.57)	17.2 (7.12)	**0.01**
**Mortality**				
N (%)	3 (2.4%)	12 (15.6%)	15 (7.4%)	0.94
**Hospitalizations**				
N (%)	6 (4.7%)	6 (7.8%)	11 (5.4%)	0.54
**Falls**				
N (%)	5 (3.9%)	17 (22.1%)	22 (10.8%)	0.66

Abbreviations (in alphabetic order): ADL = Activities of Daily Living, CIRS = Cumulative Illness Rating Scale, GDS = Geriatric Depression Scale, HGS = Handgrip Strength; IADL = Instrumental Activities of Daily Living, MNA = Mini Nutritional Assessment, MPI = Multidimensional Prognostic Index, SD= Standard Deviation, SPPB = Short Physical Performance Battery, SPSMQ = Short Portable Mental Status Questionnaire. Bold font is used to highlight statistically significant *p*-values (*p* < 0.05).

**Table 3:** Association of dynapenia and polypharmacy with the risk (HR) of negative outcomes (mortality, falls, and hospitalizations).

Variable	HR	CI 95%	*p*-Value	HR	CI 95%	*p*-Value
	**Unadjusted**			**Adjusted**		
Dynapenia	5.61	(2.51–12.57)	**<0.001**	2.96	(1.22–7.20)	**0.016**
Polypharmacy	2.34	(1.20–4.30)	**0.006**	2.23	(1.24–4.10)	**0.007**

Abbreviations: HR = Hazard Ratio, CI = Confidence Intervals. Bold font is used to highlight statistically significant *p*-values (*p* < 0.05).

**Table 4:** Logistic regression analysis showing the association between polypharmacy at baseline and 6 months, and the occurrence of adverse outcomes.

Variable	OR	CI 95%	*p*-Value	OR	CI 95%	*p*-Value
	**Unadjusted**			**Adjusted**		
Reference	1 [reference]			1 [reference]		
Polypharmacy (Time 0)	1.53	(0.88–2.68)	0.13	0.78	(0.38–1.48)	0.43
Reference	1 [reference]			1 [reference]		
Polypharmacy (6 months)	2.28	(1.19–4.42)	**0.013**	2.63	(1.21–4.63)	**0.019**

Abbreviations: OR = Hazard Ratio, CI = Confidence Intervals. Bold font is used to highlight statistically significant *p*-values (*p* < 0.05).

**Table 5:** Association of specific medications commonly used in older people with the risk (HR) of negative outcomes (mortality, falls, hospitalizations).

Variable	HR	CI 95%	*p*-Value
No use of this kind of medication (reference)	1 [reference]		1 [reference]
Antipsychotics/Mood stabilizers	3.27	(1.43–7.50)	**0.005**
Benzodiazepines/Z-drugs	2.93	1.(27–6.74)	**0.012**
Antidepressants	1.87	(0.44–7.91)	0.392

Abbreviations: HR = Hazard Ratio, CI = Confidence Intervals. Bold font is used to highlight statistically significant *p*-values (*p* < 0.05).

## Data Availability

The datasets generated and/or analyzed during the current study are available from the corresponding author upon request.

## Abbreviations

The following abbreviations are used in this manuscript:

ADL	Activities of Daily Living
CIRS	Cumulative Illness Rating Scale
GDS	Geriatric Depression Scale
HGS	Handgrip Strength
IADL	Instrumental Activities of Daily Living
MNA	Mini Nutritional Assessment
MPI	Multidimensional Prognostic Index
SPPB	Short Physical Performance Battery
SPSMQ	Short Portable Mental Status Questionnaire
